# Regiomontan: A Regional High Precision Ionosphere Delay Model and Its Application in Precise Point Positioning

**DOI:** 10.3390/s20102845

**Published:** 2020-05-16

**Authors:** Janina Boisits, Marcus Glaner, Robert Weber

**Affiliations:** Research Division Higher Geodesy, Department of Geodesy and Geoinformation, TU Wien, 1040 Vienna, Austria; janina.boisits@geo.tuwien.ac.at (J.B.); robert.weber@geo.tuwien.ac.at (R.W.)

**Keywords:** ionosphere delay modeling, GNSS signal delay correction, GNSS positioning, Precise Point Positioning, ionospheric constraint, convergence

## Abstract

Propagation delays of GNSS signals caused by the ionosphere can range up to several meters in zenith direction and need to be corrected. Geodetic receivers observing at two or more frequencies allow the mitigation of the ionospheric effects by forming linear combinations. However, single frequency users depend on external information. The ionosphere delay model Regiomontan developed at TU Wien is a regional ionospheric delay model providing high accuracy information with a latency of only a few hours. The model is based on dual-frequency phase observations of a regional network operated by EPOSA (Echtzeit Positionierung Austria) and partners. The corrections cover a geographical extent for receiver positions within Austria and are provided in the standardized IONEX format. The performance of Regiomontan as well as its application in Precise Point Positioning (PPP) were tested with our in-house PPP software raPPPid using the so-called uncombined model with ionospheric constraint. Various tests, e.g., analyzing the coordinate convergence behavior or the difference between estimated and modeled ionospheric delay, proving the high level of accuracy provided with Regiomontan. We conclude that Regiomontan performs at a similar level of accuracy as IGS final TEC maps, but with explicitly reduced latency.

## 1. Introduction

The upper part of the atmosphere ranging from approximately 60 to 2000 km (e.g., [1,2]) is referred to as ionosphere. In this layer, the absorption of certain frequencies of the solar radiation (mostly Ultra Violet (UV) radiation) results in a separation of neutral gas atoms or molecules into free electrons and ions. This process is called ionization. The level of ionization primarily depends on the solar activity and the geomagnetic field [2]. The free electrons and ions interact with microwaves, such as the signals of Global Navigation Satellite Systems (GNSS), and affect their propagation velocity, known as ionosphere refraction. The resulting ionospheric delay (or advancement in case of carrier-phase observations) ranges up to several meters in zenith direction, depending on latitude and longitude in a sun-fixed coordinate system. Thus, the ionosphere refraction needs to be considered in GNSS and other space geodetic techniques operating with microwave signals.

Regarding signals in the microwave band, the ionosphere is a dispersive medium. Thus, the ionosphere refraction can be mitigated using a linear combination (LC) of two or more observables of the same type (e.g., carrier-phase), but at different frequencies (see Section 2). However, a great number of single-frequency users depends on external information to correct the ionospheric refraction. For this purpose, regional and global ionosphere models are provided.

A simple model for correcting about 50% of the ionosphere refraction is the Klobuchar model [3]. The model algorithm yields a time delay which can be converted to a range error by multiplying it with the speed of light. The Klobuchar model describes the global behavior of the ionosphere using a constant value during the night and a cosine term during the day. Since in many parts of the world the maximum ionization is reached around two hours after the culmination of the sun [3], a phase shift of 14 h is applied so that the maximum value of the cosine term occurs at 14:00 local time. The amplitude and period of the cosine term are computed using a third-order series expansion, each. In this way, the Klobuchar model is fully described by the eight coefficients of the series expansions. These coefficients are broadcasted daily via the Global Positioning System (GPS) navigation message [4] and, thus, are available in real-time.

The approach of the NeQuick model [5,6], which emerged from the DGR profiler (named after the authors Di Giovanni and Radicella) [7], is to describe the electron density as well as electron density profiles for arbitrary signal paths. The model is split into a bottomside formulation below the height of maximum electron density and into a topside formulation above that height. In the current version of the model, NeQuick 2 [8], the bottomside formulation can be computed as a sum of semi-Epstein layers (ref) and the topside formulation is expressed as a single semi-Epstein layer. The key input parameters are position, time, and solar flux [8]. By integrating the resulting electron density profile, the total electron content (TEC) is obtained and can be converted to a range error (see Section 2).

Hoque et al. [9] addressed the shortcomings of the NeQuick model, meaning primarily the computational cost. The authors presented the Neustrelitz TEC Models (NTCM) working with the same input arguments as the NeQuick model that are broadcast in the Galileo navigation message. They reported a better performance of NTCM than NeQuick when compared to the reference values from the International GNSS Service (IGS) [10]. Furthermore, processing the NTCM is on average 65 times faster than processing NeQuick.

Most commonly used ionosphere delay models consider only the first-order term of the propagation delay. However, there are numerous studies that investigate the impact of the higher order terms of the ionospheric delay (e.g., [11,12]). Already in 2007, Hoque and Jakowski [12] concluded that the importance of modeling higher-order terms will increase with the upcoming of high precision GNSS and that they cannot be neglected for achieving millimeter-level accuracy. In a recent study, Aragon-Angel et al. [13] conducted an analysis of the remaining error between the most accurate computation of I2+ terms and a simplified modeling. They found that the second-order term I2 can reach up to 5 mm in zenith direction. Other effects, such as geometric bending, can also range up to several millimeters and reach the centimeter-level during storm conditions. A corresponding impact is found in GNSS products such as satellite orbits and satellite clocks.

Ionosphere delay models can also be derived from Precise Point Positioning (PPP) algorithms utilizing the ionosphere-free LC. Rovira-Garcia et al. [14] calculated a global real-time model using PPP by separating the ionospheric delay after fixing the ambiguities. The delay is estimated every 5 min with an accuracy better than 1 TEC Units. The resulting ionospheric model allows a much shorter convergence time for 2-frequency GNSS users and an improvement in positioning for single-frequency users. Hernández-Pajares et al. [15] presented a concept for the future use of mass market single-frequency receivers for sounding the ionosphere. In their study, the authors form the ionospheric graphic combination (IG) where the non-frequency dependent terms cancel. Once calibrated with external ionospheric information or in-situ dual-frequency observations, the receiver can be used for monitoring changes in TEC relative to previous measurements with the same geometry (one sidereal day for GPS). Following this approach, single-frequency receivers could contribute to monitoring the ionosphere.

In a very recent study, Li et al. [16] presented real-time global ionospheric maps (RT-GIMs) based on the IGS real-time service to correct for the ionosphere. The ionospheric information is derived from multi-frequency and multi-GNSS observations by forming the geometry-free linear combination. As background ionospheric information, a two-day predicted GIM is introduced additionally. The authors reported that the RT-GIMs perform only slightly worse than the respective post-processed GIMs. Unfortunately, these results are not further integrated into our own study, because they were published after we concluded our experiment.

The Regiomontan model presented in this paper aims to provide a high precision ionosphere delay information for single-frequency users with a latency as short as possible and independent from PPP. Regiomontan was developed at TU Wien as part of the correspondent project funded by the Austrian Research Promotion Agency (Österreichische Forschungsförderungsgesellschaft (FFG)) [17,18]. After completion of the project, the software algorithm is now set up to a near real-time service as part of the Vienna VLBI and Satellite Software (VieVS) [19]. Since Regiomontan is a regional model with a geographical extent designed for receiver locations in Austria, the computational effort is very small compared to global models and the data transfer of the hourly observation files is completed a few minutes after every full hour. These advantages result in a latency of only 3–4 h delivering a precision competitive to the final ionospheric maps of the IGS [10].

To demonstrate the performance of Regiomontan, we present its application in PPP in comparison to ionosphere models of the highest quality and the ionosphere free linear combination. PPP is one of the most promising processing techniques for GNSS data and was firstly proposed by Zumberge et al. [20]. The coordinate convergence time is well known as the major concern of this method and therefore a major topic in scientific research. The uncombined model has received more and more attention in the last few years because it relies on the raw GNSS observations and therefore keeps the noise of the raw measurements. This approach can easily be applied to any number of frequencies and opens up new possibilities [21]. In contrary to the classical PPP model which relies on the ionosphere-free LC, the ionospheric delay has to be considered in the uncombined model and it is possible to introduce ionospheric delays from external ionosphere models [22]. Due to the limited accuracy of such models, it is not sufficient to just correct the GNSS measurements with modeled ionospheric delays as this would bother the coordinate accuracy of the PPP solution [23]. Therefore, constraining the ionosphere with so-called ionospheric pseudo-observations has proven as the most promising approach [22,24]. In addition, tropospheric constraints have proven their usefulness in [25,26] alone or in addition to the ionospheric constraint (“atmospheric constraint”). The weighting of the ionospheric pseudo-observations has proven to have a direct influence on the coordinate convergence time [25]. The use of ionospheric pseudo-observations in all epochs with a constant variance as described in [27] has not proven to be optimal [24]. Therefore, a linear decrease of the ionospheric constraint is presented and used in this contribution based on the approach of [28].

An introduction to the theoretical background of ionospheric delay estimation is given in Section 2. Section 3 presents the Regiomontan model algorithm and its parameters. The application of Regiomontan to PPP using the uncombined model with ionospheric constraint is described in Section 4. The results of the model validation and its use in PPP are discussed in Section 5. Section 6 provides conclusions.

## 2. Estimating the Ionospheric Delay from GNSS Observations

When considering only the first-order term, the relation between electron density and ionosphere refraction can be expressed as (e.g., [29,30,31]):

(1) $$dIon=\frac{40.31}{f^{2}}STEC=\frac{40.31}{f^{2}}\int N_{e}\left(s\right)ds$$

where dIon is the slant signal delay in meters, *f* is the frequency of the electromagnetic wave, and $N_{e}$ is the electron density along the signal path *s*. The constant 40.31 is calculated from the physical quantities electron charge, permittivity of free space, and electron mass [31]. The slant total electron content STEC is the integrated electron density and is equivalent to the amount of free electrons in a cylinder with a cross section of 1 m^2^ and a height of the slant signal path [31]. The STEC is measured in total electron content units (TECU), where 1 TECU accounts for 10^16^ electrons/m^2^.

The slant signal delay appears in the GNSS observation equation for code observations as well as phase observations, but with opposite sign. The GNSS observation equations (in m) can be written as:

(2) $$P_{i}=\rho +c\left(dt_{R}-dt^{S}\right)+dTrop+dIon_{i}+c\left(B_{R}+B^{S}\right)+\varepsilon$$

(3) $$L_{i}=\rho +c\left(dt_{R}-dt^{S}\right)+dTrop-dIon_{i}+\lambda_{i}b+\varepsilon$$

with the code observable $P_{i}$ and the phase observable $L_{i}$ on frequency *i*, the geometric distance between satellite and receiver ρ, receiver clock error $dt_{R}$ and satellite clock error $dt^{S}$, both multiplied by the speed of light *c*. dTrop and $dIon_{i}$ denote the tropospheric and the ionospheric delays and $B_{R}$ and $B^{S}$ are the code hardware delays of receiver and satellite, respectively. $\lambda_{i}b$ is the ambiguity term in length units, including the integer term as well as the hardware biases, and ε comprises random and other negligible errors.

The ionosphere is a dispersive medium for microwave signals, as Equation (1) indicates. Thus, an ionosphere-free linear combination (IF LC) of signals at different wavelengths can be used to eliminate the dispersive component of the ionosphere refraction. The remaining higher-order terms account for approximately 0.1% of the total delay [31] and can be neglected in most cases.

Conversely, the ionospheric delay can be estimated by forming the geometry-free LC for code observations P4 (Equation (4)) or for phase observations L4 (Equation (5)), and further converted to the STEC using Equation (1).

(4) $$P4=P1-P2=\left(1-\frac{f_{1}^{2}}{f_{2}^{2}}\right)dIon+c\left(DCB_{R}+DCB^{S}\right)$$

(5) $$L4=L1-L2=-\left(1-\frac{f_{1}^{2}}{f_{2}^{2}}\right)dIon+\lambda b_{4}$$

where P1 and P2 are the code measurements and L1 and L2 are the phase measurements at the carrier frequencies $f_{1}$ and $f_{2}$, respectively. $DCB_{R}$ and $DCB^{S}$ denote the frequency-dependent differential code biases (also referred to as inter-frequency biases) for receiver and satellite, and $\lambda b_{4}$ is the ambiguity term (in m) for the given linear combination.

The highest electron density occurs in the so-called F2 layer with a peak density height ranging from 250 to 500 km [32]. Thus, a single layer model (SLM) [33] can be used to approximate the ionospheric effect, where all free electrons are assumed to be condensed on an infinitesimally thin layer. The signal path intersects the single layer in the ionospheric pierce point (IPP). Applying the SLM mapping function (MF) given in Equation (6) [33] to the STEC yields the vertical total electron content VTEC at the location of the IPP.

(6) $$MF\left(\varepsilon \right)=\frac{1}{\sqrt{1-{\left(\frac{R}{R+H}cos\varepsilon \right)}^{2}}}$$

with the elevation angle ε of the observation, *R* the mean Earth radius, *H* the height of the single layer, and STEC=MF(ε)·VTEC.

The global behavior of the ionosphere can then be expressed by a spherical harmonics (SH) expansion of the VTEC [33]. Evaluating the SH expansion on a geographical grid and at a given epoch delivers a TEC map, which is usually distributed in the IONospheric EXchange (IONEX) format [34]. The IGS provides two products using the IONEX format. First, the rapid ionospheric TEC grid is published with a latency of less than 24 h with an accuracy of 2–9 TECU, and, second, the final ionospheric TEC grid with a latency of about 11 days and an accuracy of 2–8 TECU [10].

## 3. Development of a Regional High Precision Ionosphere Delay Model

### 3.1. Taylor Approximation of the Single-Layer Model

The SLM forms the theoretical basis of the Regiomontan model. The VTEC values of the single layer are regionally approximated using a two-dimensional Taylor expansion of degree two, where the height of the layer is set to 350 km:

(7) $$\begin{array}{cc} VTEC_{IPP}= & VTEC_{0}+\frac{dVTEC}{d\varphi }\Delta \varphi_{IPP}+\frac{dVTEC}{d\lambda }\Delta \lambda_{IPP}\\ \; & +\frac{1}{2}\frac{d^{2}VTEC}{d\varphi^{2}}\Delta \varphi_{IPP}^{2}+\frac{d^{2}VTEC}{d\varphi d\lambda }\Delta \varphi_{IPP}\Delta \lambda_{IPP}+\frac{1}{2}\frac{d^{2}VTEC}{d\lambda^{2}}\Delta \lambda_{IPP}^{2} \end{array}$$

with $\Delta \varphi_{IPP}=\varphi_{IPP}-\varphi_{P_{0}}$ and $\Delta \lambda_{IPP}=\lambda_{IPP}-\lambda_{P_{0}}$

$VTEC_{IPP}$ denotes the vertical TEC derived from GNSS observations at the respective IPP. $VTEC_{0}$ is the vertical TEC at the center of the Taylor expansion $P_{0}$. Together with the first and second derivatives of VTEC with respect to geographical latitude φ and geographical longitude λ, it forms the six parameters of the Regiomontan model.

**Figure 1:**
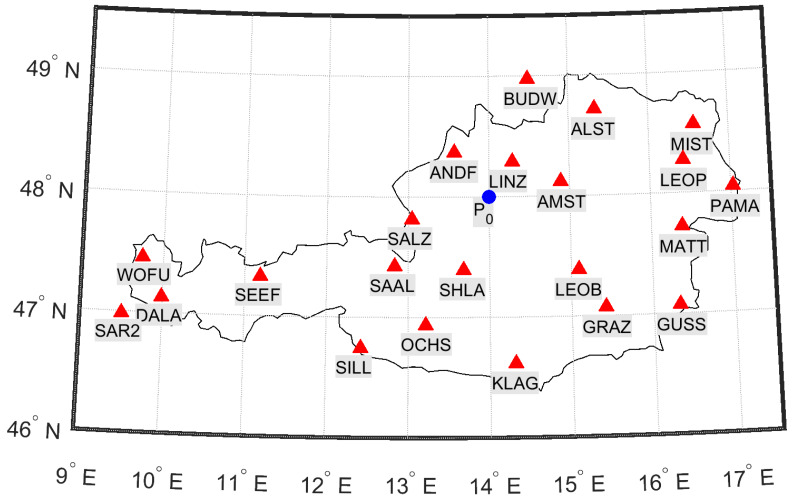
GNSS station network used for processing the Regiomontan model. Red triangles depict the permanent reference stations selected for this purpose. The blue dot indicates the center of the Taylor expansion $P_{0}$.

The center of the Taylor expansion $P_{0}\left(\varphi_{P_{0}},\lambda_{P_{0}}\right)$ is located in the center of Austria at 48° latitude and 14° longitude (Figure 1). The IPP coordinates $\left(\varphi_{IPP},\lambda_{IPP}\right)$ are calculated based on the observation geometry using satellite coordinates extracted from the IGS ultra-rapid orbits [10]. The station network selected for processing the Regiomontan model comprises 22 permanent GNSS reference stations in Austria and neighbouring countries (Figure 1) operated by EPOSA (Echtzeit Positionierung Austria) [35] and partners. The observation files include code and phase measurements with an update rate of 1 s.

### 3.2. Parameter Estimation Using Code-Leveled Carrier-Phase Observations

To estimate the model parameters $VTEC_{0}$, as well as first and second derivatives of VTEC (see Equation (7)), the $VTEC_{IPP}$ values need to be computed. Thus, the geometry-free LC for 2-frequency phase observations (Equation (5)) is formed, yielding the dispersive component of the ionospheric delay dIon. At the moment, Regiomontan is based on GPS observations only. The reasons for this are mainly the satellites and signals tracked by EPOSA stations, but also the availability of auxiliary data, such as satellite orbits or DCBs. The ambiguity term $\lambda b_{4}$ of Equation (5) is eliminated by applying a leveling approach following Banville et al. [36]. For this purpose, a mean range bias $\hat{\lambda b_{4}}$ between the geometry-free LC for code observations (Equation (4)) and for phase observations (Equation (5)) is estimated for every individual satellite arc:

(8) $$\hat{\lambda b_{4}}=\langle P4+L4-c\left(DCB_{R}+DCB^{S}\right)\rangle$$

The obtained ionospheric delay dIon can be converted to STEC values using Equation (1) and, subsequently, to VTEC values by applying the SLM Mapping Function given in Equation (6).

IGS products are employed to account for satellite Differential Code Biases $DCB^{S}$ in Equation (8). The station Differential Code Biases $DCB_{R}$ for the Regiomontan station network are estimated weekly, solving Equation (4) for the $DCB_{R}$ of every station. dIon is extracted from the final ionospheric TEC grids provided by the IGS [10] and the $DCB_{R}$ values are estimated in a least squares adjustment combining all carrier-smoothed code observations of one day.

**Figure 2:**
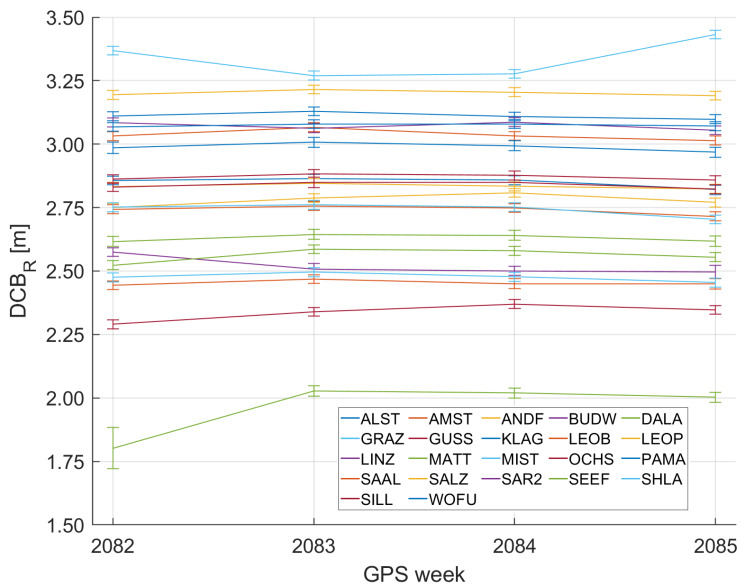
Station DCB values $DCB_{R}$ of the Regiomontan station network over four consecutive GNSS weeks in December 2019. The $DCB_{R}$ values mostly vary only a few centimeters during this period. The error bars indicate the standard deviation of the least squares adjustment.

Figure 2 illustrates the behavior of $DCB_{R}$ during four consecutive GPS weeks in December 2019. The $DCB_{R}$ of each station is quite stable and mostly varies only a few centimeters during one month. Thus, the weekly estimation of $DCB_{R}$ values is found to be appropriate for this purpose.

Based on Equation (7), the Regiomontan model parameters are estimated in a least squares adjustment for every full hour *t* of one day, bundling all observations of the interval t±30 min for the computation. Exceptions are the first (00:00 UTC) and the last (24:00 UTC) epochs of the day, where the intervals t+30 min and t−30 min are used, respectively.

**Figure 3:**
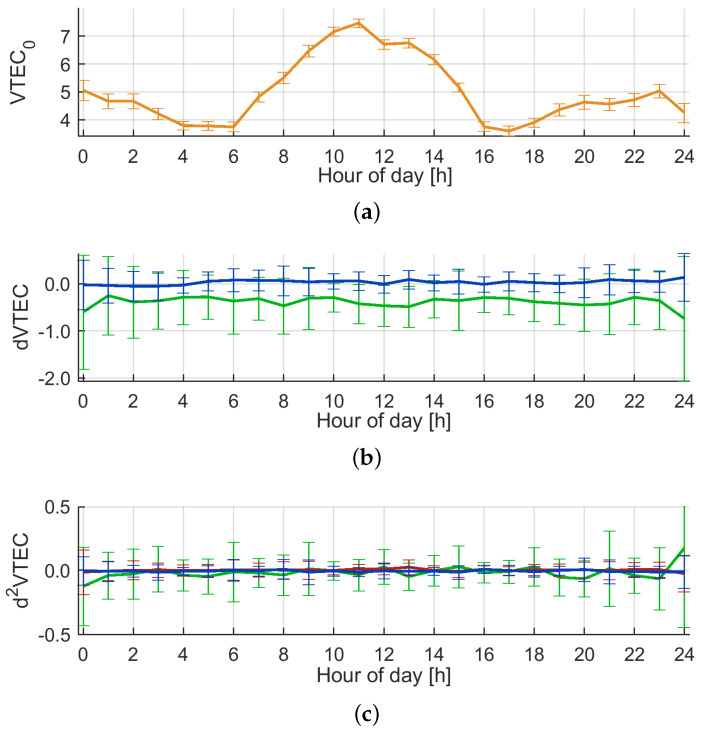
Behavior of the Regiomontan model parameters on doy 335, 2019: (**a**) The top panel depicts the behavior of $VTEC_{0}$ in TECU. The error bars illustrate the formal errors of the least squares adjustment magnified by the factor of 10${}^{2}$. (**b**) The middle panel depicts the behavior of the first derivatives of VTEC with respect to latitude (green) and longitude (blue) in TECU/rad. Formal errors are magnified by the factor of 10${}^{3}$. (**c**) The bottom panel depicts the behavior of the second derivatives of VTEC with respect to latitude (green), longitude (blue), and the mixed term (red) in TECU${}^{2}$/rad${}^{2}$. Formal errors are magnified by the factor of 10${}^{3}$.

The results of the parameter estimation for 1 December 2019, day of year (doy) 335, are illustrated in Figure 3. The top panel depicts the vertical TEC at $P_{0}$, $VTEC_{0}$, with typically low values around 4 TECU during night and a maximum of 7.5 TECU around noon UTC. The formal errors for $VTEC_{0}$ from the least squares adjustment are at the order of 10${}^{-3}$ TECU. The formal errors in Figure 3a are magnified by the factor of 10${}^{2}$ for better visibility. The unrealistic small formal errors of the model parameters can be explained by the high correlation between the observations with an update rate of 1 s. Furthermore, errors introduced by the mapping function were neglected in this study.

Figure 3b illustrates the results for the first derivatives of VTEC with respect to latitude φ and longitude λ. The first derivative with respect to latitude is negative due to a generally higher ionization in the equatorial regions, while the first derivative with respect to longitude is very low during winter. This originates in the generally low ionization at mid-latitudes during winter. In summer, the first derivative with respect to longitude clearly reflects the passing of the peak ionization with the sun. The formal errors from the least squares adjustment are at the level of 10${}^{-4}$ TECU/rad. Figure 3c depicts the second derivatives of VTEC. One can notice here that the second derivative with respect to latitude is less well determined than the one with respect to longitude due to the station network geometry. The formal errors from the least squares adjustment are at the level of 10${}^{-4}$ TECU${}^{2}$/rad${}^{2}$.

### 3.3. Specifications of the Output Files in IONEX Format

For easier application of the Regiomontan model, the output parameters are converted to the standardized IONEX format [34]. Thus, the Taylor expansion is evaluated on a regular grid with a graphical extent designed for user locations within Austria. The specifications of the resulting TEC maps provided in the Regiomontan IONEX files are listed in Table 1.

Since the code-leveling approach described in Section 3.2 is a post-processing technique, the Regiomontan model cannot be provided in real-time. However, the model algorithm requires only low computational cost and the file transfer is usually completed around 10 min after every full hour. Exploiting these advantages, the model processing is finished around 04:30 CET/CEST. Thus, the Regiomontan IONEX files can be requested via the EPOSA website [35] in the early morning on the following day. In this way, a high precision ionosphere delay model with a high spatial resolution is provided with a latency of only a few hours.

## 4. Applying the Regiomontan Model to Precise Point Positioning

PPP can be characterized through the use of precise satellite products (e.g., orbits, clocks, and biases), accurate observation models, and sophisticated algorithms, which all are applied on the observation of a single GNSS receiver. In that way a coordinate accuracy at the dm to cm level for the float solution and at the cm to mm level for the fixed solution can be achieved. Normally, a Kalman filter is used to estimate the coordinates and additional parameters. PPP is an absolute positioning method and in contrary to relative positioning methods (e.g., RTK) no nearby reference station or network are needed. On the other hand, PPP has a non-negligible convergence time, and, to make PPP more competitive against other high-precision GNSS positioning techniques, scientific research focuses on diminishing convergence periods. As the classical PPP approach using the ionosphere-free linear combination (IF LC) is limited in that regard, the uncombined model based on the raw GNSS observation equations has become quite trendy in the last few years.

### 4.1. Classical PPP Model

The classical PPP approach uses observations on two frequencies to build the IF LC which removes the first-order ionospheric delay. As terms of higher order of the ionosphere can be neglected for PPP, the ionospheric delay is completely eliminated from the mathematical model. Therefore, in this formulation, no information about the ionospheric delay can be obtained or be introduced from, e.g., an ionosphere model such as Regiomontan. Building the IF LC increases the observation noise, which elongates the coordinate convergence time [22] and is not invertible [37]. As two frequencies are essential to build the LC, it is not possible to use this model with observations on one frequency only. Furthermore, it is not clear how to extend it against the background of modern GNSS signals on three or more frequencies. Neither a 3-frequency IF LC nor the use of two 2-frequency IF LCs is a sensible approach.

(9) $$P_{IF}=\rho -c\left(dt_{R}^{gps}+\delta t_{R}^{g}\right)+dTrop^{wet}+\varepsilon$$

(10) $$L_{IF}=\rho -c\left(dt_{R}^{gps}+\delta t_{R}^{g}\right)+dTrop^{wet}+\lambda_{IF}b+\varepsilon$$

Equations (9) and (10) show the observation equations of the classical PPP model for the code $P_{IF}$ and phase measurement $L_{IF}$ in meters where the subscript ${}_{IF}$ stands for ionosphere free linear combination [22]. In comparison to the GNSS observation in Equations (2) and (3), the satellite clock error $cdt^{s}$ is omitted because of the use of precise satellite products. For the same reason, no satellite orbit error is included. The ionospheric delay $dIon_{i}$ is removed (IF LC) and the tropospheric delay dTrop is split into a hydrostatic part (modeled and omitted) and a wet part $dTrop^{wet}$, which is estimated. A receiver clock offset $\delta t_{R}^{g}$ to the GPS receiver clock error $cdt_{R}^{gps}$ is added and estimated for each processed GNSS other than GPS. The estimated receiver coordinates are covered in the geometrical distance ρ term. The phase ambiguities *b* also contain constant hardware biases and are estimated as real valued numbers therefore. To solve the ambiguities to their integer values, these hardware biases have to be considered for the satellites and the receiver for which different approaches exist [38]. Thanks to the IF LC, no receiver DCBs have to be considered because the receiver code biases get converted into a constant bias, which is absorbed by the estimation of the receiver clock error. Some error terms which have to be considered in PPP (e.g., phase wind-up, phase center variations, etc.) are modeled and are therefore also not included in the observation equations [39].

### 4.2. Uncombined PPP Model with Ionospheric Constraint

The so-called uncombined model applies the raw GNSS observation (Equations (2) and (3)). Therefore, the observation noise of the original measurements is kept and not increased through the coefficients of a LC, which improves the convergence [21]. Furthermore, the ionospheric delay is not eliminated from the mathematical model [37] and estimated on the first frequency of the corresponding GNSS (e.g., GPS L1 and Galileo E1) for each satellite. To scale the ionospheric delay to other frequencies, the coefficient calculated with Equation (11) is used in the design matrix. In this model, the receiver code biases $DCB_{1i}$ have to be considered and estimation is a reasonable way [40]. It would also be possible to correct the receiver DCBs with known values. In addition, satellite DCBs have to be applied properly to get consistency [21] when satellite clock products calculated with the IF LC are used.

(11) $$dIono_{1}=\frac{f_{1}^{2}}{f_{i}^{2}}\cdot dIono_{i}$$

(12) $$P_{i}=\rho -c\left(dt_{R}^{GPS}+\delta t^{g}\right)-DCB_{1i}+dTrop^{wet}+\frac{f_{1}^{2}}{f_{i}^{2}}\cdot dIono_{1}+\varepsilon$$

(13) $$L_{i}=\rho -c\left(dt_{R}^{GPS}+\delta t^{g}\right)-DCB_{1i}+dTrop^{wet}-\frac{f_{1}^{2}}{f_{i}^{2}}\cdot dIono_{1}+\lambda_{i}b_{i}+\varepsilon$$

(14) $$dIono_{pseudo}=dIono_{1}+\varepsilon$$

Equations (12) and (13) present the observation equations in meters for the code $P_{i}$ and phase $L_{i}$ measurement of the uncombined model. In comparison to the observation equations of the classical PPP model in Equations (9) and (10), it becomes clear that this model can easily be extended to any number of frequencies. Moreover, it is suitable for single-frequency data and is much more flexible in terms of missing observations (e.g., GPS L5). This is practical in view of the current GNSS constellations, and it even is possible to process a different number of frequencies for each GNSS.

Through adding modeled ionospheric delays to each satellite on the first frequency as ionospheric pseudo-observations to the observation model (Equation (14)), we get the uncombined model with ionospheric constraint. This strategy is necessary to shorten the convergence time because the ionospheric delay and receiver code biases are fully correlated and a rank rank deficiency occurs between these parameters [41]. In the case of estimating both quantities without ionospheric constraint in parallel, it is not possible to separate them. This will result in a long convergence time [40] and non-sensible (e.g., negative) values for the ionospheric delay and receiver DCBs, which are contaminated from each other.

A crucial point when using ionospheric pseudo-observations is their observation variance and weighting in the Kalman filter. If the ionospheric pseudo-observations are weighted too high, the estimated ionospheric delay will be dragged too much to the ionosphere model and its imperfection (“over constraining”). However, also too low weights (“under constraining”) will degrade convergence [25]. Following Cai et al. [28] and the results of Ning et al. [24], where a time-varying weighting scheme has proven its advantage over other approaches, the variance of the ionospheric constraint is increased over time with a linear approach in this study, and, after a certain point in time $t_{end}$, ionospheric pseudo-observations are no longer used. This is based on the idea that the ionospheric constraint enhances the estimation of the ionospheric delay especially at the beginning of the processing. Therefore, a linear interpolation as presented in Equation (15) is used.

(15) $$\sigma_{iono}^{2}\left(t\right)=\sigma_{iono,0}^{2}+\frac{\sigma_{iono,end}^{2}-\sigma_{iono,0}^{2}}{t_{end}}\cdot t$$

where *t* is the time since the start of the solution and $\sigma_{iono}^{2}\left(t\right)$ is the variance of the ionospheric pseudo-observations at *t*. At the initial epoch of the solution, the ionospheric pseudo-observations are weighted with variance $\sigma_{iono,0}^{2}$ and will be altered towards the variance $\sigma_{iono,end}^{2}$ at $t_{end}$. Therefore, the parameters $\sigma_{iono,0}^{2}$, $t_{end}$, and $\sigma_{iono,end}^{2}$ define the strength of the ionospheric constraint and how fast it is released. Another strategy can be found in [42] where the variances of the ionospheric constraint are adjusted according to the sum of the quadratic forms of weighted residuals.

In our work, the ionospheric delay is calculated from the Regiomontan model, which is provided in the IONEX format [34]. The value of the mapping function is calculated according to Equation (6) and the latitude and longitude for the IPP are calculated. Afterwards, the VTEC is interpolated in space and time utilizing the “consecutive rotated maps” algorithm [34], which performs best in [26]. Then, the ionospheric delay is calculated with Equation (1).

## 5. Results

### 5.1. Validating Regiomontan via Ionosphere-Free Linear Combination

In a first step, Regiomontan was validated by applying model delays to carrier-smoothed pseudo-range observations P1, in the following referred to as test values or P1+dIon. These test observations were compared to the ionosphere-free LC of carrier-smoothed pseudo-range observations P3, in the following also referred to as reference values, by forming the residuals (P1+dIon)−P3.

**Figure 4:**
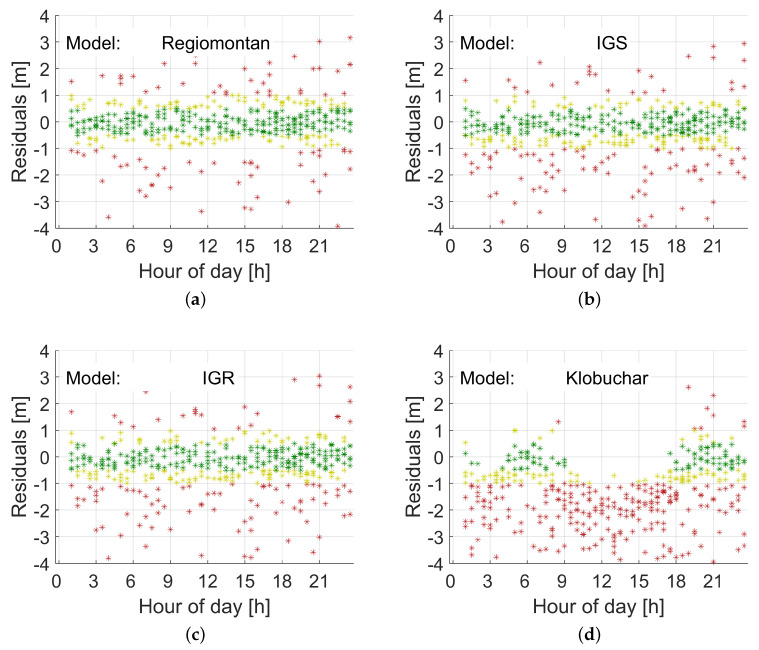
Range residuals for station WOFU on doy 153, 2019. Green indicates residuals smaller than 0.5 m, yellow residuals smaller than 1 m, and red residuals larger than 1 m: (**a**) Regiomontan; (**b**) IGS final TEC grid (IGS); (**c**) IGS rapid TEC grid (IGR); and (**d**) Klobuchar. The Klobuchar model overestimates the ionospheric delay during the daytime. The other models yield very similar results. All observations down to an elevation angle of 5° are included.

**Figure 5:**
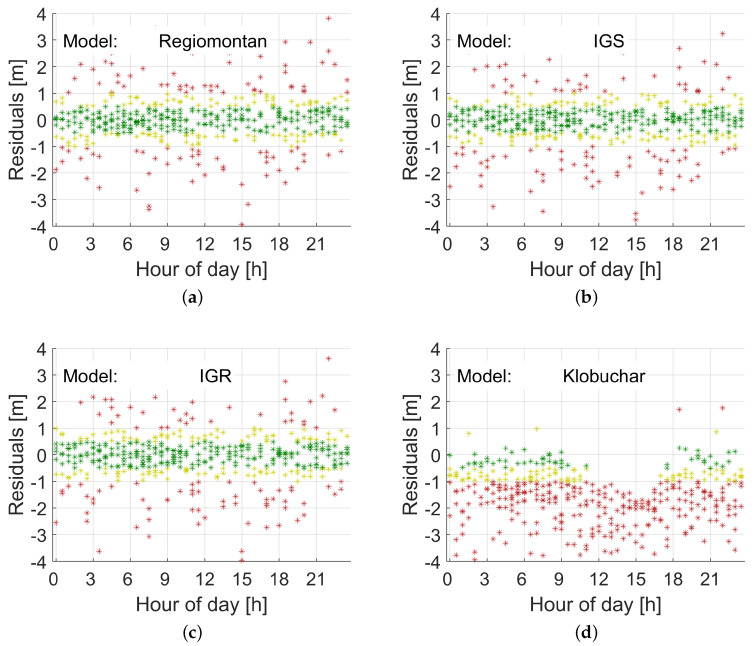
Range residuals for station WOFU on doy 335, 2019. Green indicates residuals smaller than 0.5 m, yellow residuals smaller than 1 m, and red residuals larger than 1 m: (**a**) Regiomontan; (**b**) IGS final TEC grid (IGS); (**c**) IGS rapid TEC grid (IGR); and (**d**) Klobuchar. All four models yield the same level of accuracy in winter as they do in summer. All observations down to an elevation angle of 5° are included.

Figure 4 and Figure 5 illustrate the results for station WOFU. This station is located in the west of Austria (see Figure 1) at the periphery of the station network, where the impact of the first and second derivative terms of VTEC (see Equation (7)) is strongest. Figure 4 demonstrates the results for a day in summer, doy 153 (2 June), in 2019, while Figure 5 the results for a day in winter, doy 335 (1 December), in 2019. The results for Regiomontan are compared to those applying the IGS final TEC grid (IGS), the IGS rapid TEC grid (IGR), and the Klobuchar model. The latter clearly overestimates the ionospheric delay, while the IGS products perform comparably to the Regiomontan model.

Table 2 lists the statistics of the residuals for each of the four models for GPS Week 2056 and GPS Week 2082. Observations down to an elevation angle of 5° are considered. The results for GPS Week 2056 are generally a few percent worse, because a software change at station BUDW was not yet considered in the DCB calculation. Furthermore, no IGS rapid TEC grids (IGR) for the days doy 336–339 of GPS Week 2082 were available. The difference between IGS and IGR is very small, while Regiomontan performs best. The statistics for the Klobuchar model confirm the results illustrated in Figure 4 and Figure 5.

### 5.2. PPP Results with Regiomontan

In this section, the Regiomontan and for comparison also other high-quality ionosphere models are introduced as background models in our uncombined PPP process. Therefore, the final global ionosphere models (GIM) of the International GNSS Service (IGS) and the Center of Orbit Determination in Europe (CODE) were chosen. The coordinates of the different PPP solutions were assessed in terms of convergence time and accuracy. Additionally the difference between the estimated and modeled ionospheric delays was studied and the values of the estimated receiver DCBs were investigated.

Regiomontan is an ionospheric model calculated from regional GNSS data and therefore only stations in Austria were chosen for testing. The EUREF stations GRAZ, PFA3, LINZ, SBG2, and TRF2 and two one-month periods in June and December 2019 were selected to cover seasonal different ionospheric conditions. Be aware that the EUREF station GRAZ, which is used here, is different from station GRAZ of the EPOSA network, which was used for the calculation of the Regiomontan model. The PPP calculations were performed with our in-house software raPPPid, which is part of the Vienna VLBI and Satellite Software (VieVS PPP). Table 3 shows an overview of the processing settings. A reset of the PPP solution was performed to every full hour. Without taking data gaps into consideration, this results in 7320 so-called convergence periods, which is the time between two resets of the solution, for the two selected months.

For the uncombined model with ionospheric constrained ionospheric pseudo-observations from Regiomontan (REGIO), the final IGS GIM or the final CODE GIM was used. To make a fair comparison with the IF LC, only two frequencies were processed also in this PPP model. As described in Section 4.2, the ionospheric delay for each satellite and the receiver DCBs qwew estimated. The initial variance of the ionospheric pseudo-observations was $\sigma_{iono,0}^{2}=0.15^{2}$ m² and the rationale for this choice is provided below. The variance was altered to $\sigma_{iono,end}^{2}=9$ m² at $t_{end}=10$ min. To ensure consistency with the code and phase observations, also an elevation dependent weighting was applied for the ionospheric pseudo-observations.

**Figure 6:**
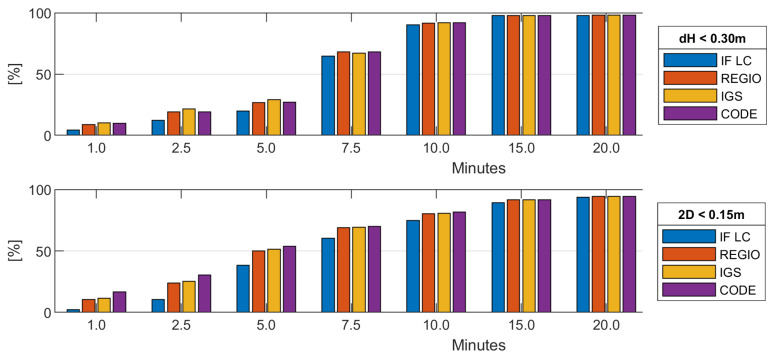
Percentage of converged solutions after [1, 2.5, 5, 7.5, 10, 15, 20] min for: the height component (**top**); and the horizontal position (**bottom**). The height of a bar corresponds to the percent of convergence periods which have reached convergence at this point in time.

Convergence is defined as the point in time when the difference to the true coordinates is under a certain threshold and stays there for the remaining convergence period. Figure 6 shows the percent of all convergence periods that converged for certain points in time for the height component (Figure 6, top) and the horizontal position (Figure 6, bottom). For the height, a threshold of 30 cm was used; for the horizontal position, a threshold of 15 cm was used. Be aware that for analysis of the coordinate convergence and accuracy the stations SBG2 and TRF2 had to be excluded. For both stations, a bias in the size of 15–20 cm in the height component between the PPP solution and the EUREF coordinates occurred. We presume that for these station a wrong antenna height is stated in the Rinex header.

As expected, the uncombined model with Regiomontan, IGS or CODE as ionospheric background shows better convergence than the IF LC, especially in the first minutes of PPP processing and the difference vanishes over time. This is a result of the higher noise of the IF LC and the use of an ionosphere background model. Comparing the different ionosphere constraints, Regiomontan performs slightly worse than the IGS model and the CODE model shows the best convergence behavior. A difference between the solutions with various ionospheric constraints can mainly be found in the first 5 min. The number of converged solution becomes increasingly similar over time as the ionosphere estimation of the PPP solution gets more reliable and more independent of the ionospheric pseudo-observations used in the first epochs and additionally the constraint is released through the increase of the variance of the ionospheric pseudo-observations.

**Figure 7:**
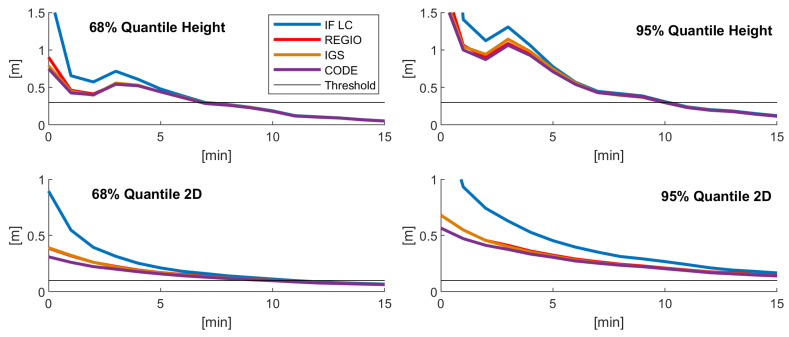
68% (**left**) and 95% (**right**) quantiles of the coordinate error in the height component (**top**) and the horizontal position error (**bottom**) for the different PPP solutions.

The above findings were confirmed when looking at the coordinate accuracy. To assess the coordinate accuracy, the mean of the 3D position error of all convergence periods for specific points in time after reset is presented in Table 4 and the 68% and 95% quantiles of the error in the height component and the horizontal position error are shown in Figure 7. In the first minutes, the uncombined model with ionospheric constraint shows a lower 3D position, height, and horizontal position error than the IF LC as the coordinates are still converging. After convergence, the position accuracy is identical. Between the different ionosphere models, only very small differences can be found. The CODE and IGS models show nearly identical results in terms of the mean 3D position error, while Regiomontan is marginally worse. Concerning the 68% and 95% quantiles, the CODE model shows a slightly better performance than the IGS and Regiomontan models. Again, the differences between the ionosphere background models decrease over time and after about 10 min no differences occur.

**Figure 8:**
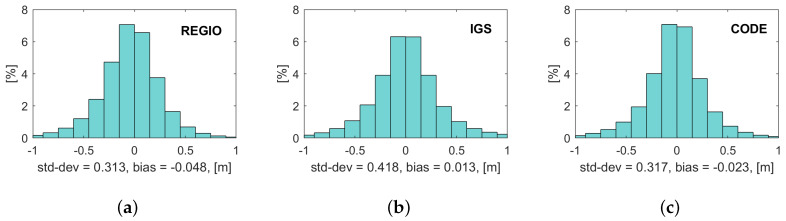
Histogram of the difference between the estimated and modeled ionospheric delay of GPS and Galileo for: (**a**) Regiomontan; (**b**) IGS; and (**c**) CODE for June and December 2019.

The difference between the modeled and estimated ionospheric delay of all processed epochs is shown in Table 5 separated for June and December and for GPS and Glonass. No seasonal dependency can be found as no significant difference occurs between the values of June and December. The difference between the modeled and estimated ionospheric delay is bigger for Glonass for all ionosphere models and also the gap between the ionosphere models. Regiomontan performs especially for the Glonass case a few percent worse than the IGS and CODE GIM and the CODE GIM is slightly superior to the IGS GIM. Figure 8 shows the histograms of the difference between the estimated and modeled ionospheric delays for both months and GNSS for the three tested ionosphere models. The Regiomontan and CODE model differences show a lower standard deviation than the IGS model but the CODE model has a smaller bias and performs therefore better. The IGS model exhibits the largest standard deviation, but the smallest bias. The noted standard deviations of about ±0.4 m are the justification for the choice of the initial variance of the ionospheric pseudo-observations $\sigma_{iono,0}^{2}=0.15^{2}$ m² to ensure a reasonable strength of the constraint.

**Figure 9:**
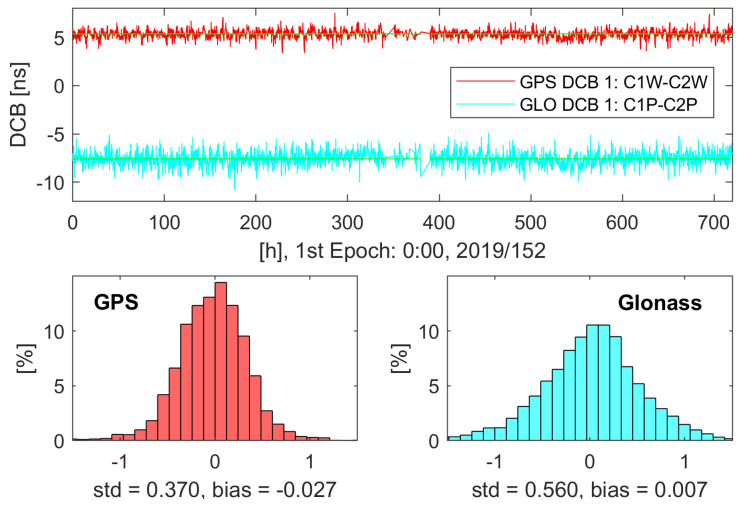
(**Top**) Estimated receiver DCBs for GPS (red) and Glonass (cyan) of the PPP solution using the uncombined model with Regiomontan as ionospheric constraint for December 2019. The CODE estimation of the receiver DCBs is shown in green. (**Bottom**) Histogram of the difference to the CODE estimation with the corresponding standard deviation (std) and bias.

The estimated receiver DCBs of the station GRAZ are shown in the upper part of Figure 9 for June 2019 using Regiomontan as ionospheric constraint. Please note that two data gaps occur on 14 and 15 June. Besides jumps at the beginning of the convergence periods, the estimated receiver DCBs are relatively constant over this month. This is the expected behavior as receiver DCBs are known to be stable over time (see Section 3.2). On the other hand, the estimated receiver DCBs show a considerable amount of noise as this parameter also absorbs model imperfections and unmodeled error sources. CODE also provides estimations for the receiver DCBs of the IGS station GRAZ. These are included as green lines in the upper part of Figure 9 and the lower part shows the appropriate histograms of the difference between the DCB estimation from the PPP solution to the CODE estimation. As can be seen, the receiver DCBs which were estimated in the PPP solution fit quite well to the estimations of CODE for both processed GNSS and the bias is negligible, which validates the Regiomontan model. The standard deviation of the Glonass receiver DCB is slightly larger than the standard deviation of the GPS receiver DCB. This can be a result of the frequency division multiple access (FDMA) which is not fully represented by estimating one receiver DCB for all Glonass satellites or perhaps the lower number of Glonass satellites.

**Figure 10:**
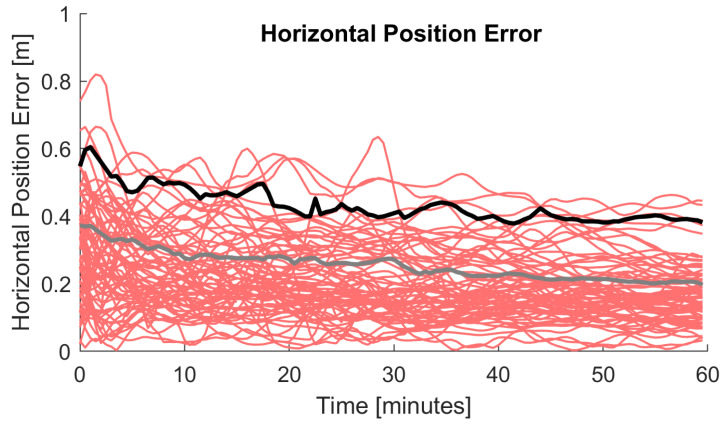
Horizontal position error for a PPP solution calculated on the next day for the station PFA3 (29–31 December 2019) using single-frequency GPS observations, the IGS ultra-rapid product, and Regiomontan. The 95% quantile is shown in black and the 68% quantile in grey.

To give an idea which PPP results can be achieved for single-frequency GPS observations as soon as the Regiomontan model is available (early morning of the next day Section 3.3), Figure 10 shows the horizontal position error over time for the station PFA3 and three arbitrary days. Again, a reset of the solution was performed each hour, which results in 120 convergence periods. In contrast to the processing settings used before (Table 3), the IGS ultra-rapid product [49] was used for the satellite orbits and clocks and GTP3 [45] was used as troposphere model because of the short latency of this PPP solution. Sub-meter accuracy is achieved instantaneously and the horizontal position error is in the size of a few decimeters.

## 6. Conclusions

The Regiomontan model presented in this paper is a regional ionospheric delay model approximating the SLM in a Taylor expansion of degree two. The parameters are calculated using the geometry-free LC of code-leveled phase observations and the model corrections are distributed in the IONEX format. Processing a regional network leads to a comparably low computational effort and, thus, Regiomontan is provided with a latency of only a few hours.

A first validation of Regiomontan was performed by forming range residuals between corrected P1 observations and the IF LC. The results affirm that Regiomontan yields the same level of accuracy as the IGS final TEC maps and, therefore, is competitive to ionosphere models of the highest quality.

The PPP tests showed that the use of Regiomontan as ionospheric background model leads to much better PPP results than the IF LC in terms of convergence and the same accuracy is achieved after convergence. We also expect that successful integer ambiguity resolution should be accelerated with Regiomontan in our further research. In the PPP solution, Regiomontan performs nearly as good as the final ionosphere models from IGS and CODE, as shown through analyzing the coordinate convergence behavior, the coordinate accuracy, and the difference between estimated and modeled ionospheric delay. No seasonal dependency was found. The shown consistency between the estimated receiver DCBs in the PPP solution and the estimated values from CODE provides another way of validation of the Regiomontan model. With more tests and novel ideas on the weighting of the ionospheric pseudo-observations, the PPP results with the uncombined model with ionospheric constraint may improve in future.

## Figures and Tables

**Table 1:** Specifications of the TEC maps provided in the Regiomontan IONEX files.

Specification	Value
# of maps	25 (00:00 UTC–00:00 UTC)
Interval	3600 s
Latitude: min, max	30°, 70°
Longitude: min, max	−20°, 45°
Spatial resolution	1° × 1°

**Table 2:** Statistics of the range residuals (P1+dIon)−P3 for all 22 stations of the Regiomontan station network. All observations down to an elevation angle of 5° are included. The results are listed for GPS Week 2056 (doy 153–159) and GPS Week 2082 (doy 335–341).

	GPS Week 2056				GPS Week 2082			
Residuals	Regiomontan	IGS	IGR	Klobuchar	Regiomontan	IGS	IGR	Klobuchar
<0.5 m	52.0%	49.3%	49.2%	24.7%	53.9%	52.5%	51.0%	13.0%
<1.0 m	76.4%	74.0%	73.9%	45.3%	79.7%	78.7%	77.6%	30.1%
<1.5 m	87.3%	85.1%	85.1%	61.0%	90.0%	89.4%	88.6%	49.0%

**Table 3:** General processing settings used for PPP tests.

Setting	Value
Stations	GRAZ, PFA3, LINZ, SBG2, TRF2
Period	June 2019, December 2019
GNSS	GPS, Glonass (weighted 1:1)
GPS Observations	L1, L2
Glonass Observations	G1, G2
Processing mode	undifferenced observations, static receiver
Observation interval	30 s, reset solution: every full hour
Raw observation noise	code = 30 cm, phase = 2 mm
Observation weighting	Elevation weighted ($sin{\left(elev\right)}^{2}$)
Cutoff angle	elevation: 5°
Satellite Orbits, Clocks and DCBs	CODE final products [43]
Satellite and receiver antenna	IGS Antex igs14.atx [44]
Troposphere model	VMF3 [45], residual ZWD is estimated
Reference coordinates	EUREF [46]
Adjustment	Kalman-Filter
Receiver clock, time offset	white noise
Phase Ambiguities	float, constant
Cycle-Slip Detection	dL1-dL2
Correction models	Phase wind-up [47], solid earth tides [48], relativistic effects

**Table 4:** Mean of 3D coordinate accuracy in centimeter for specific points in time after the last reset.

	5 min	10 min	15 min	30 min	45 min
**IF LC**	45.2	18.1	8.7	4.9	3.8
**REGIO**	39.6	16.6	8.0	4.8	3.8
**IGS**	39.1	16.5	8.0	4.7	3.8
**CODE**	39.0	16.4	7.9	4.8	3.8

**Table 5:** Statistics of the difference between the estimated and modeled ionospheric delay for the PPP solution using different ionosphere models as background model for June and December 2019.

GNSS	GPS			Glonass		
**Model**	**REGIO**	**IGS**	**CODE**	**REGIO**	**IGS**	**CODE**
Month	**June**					
<12.5 cm	42.6%	43.4%	49.7%	27.5%	30.7%	31.8%
<25 cm	69.1%	71.4%	76.6%	50.6%	56.8%	57.9%
<50 cm	89.7%	92.0%	93.4%	77.4%	85.7%	86.2%
Month	**December**					
<12.5 cm	41.2%	43.9%	47.5%	30.2%	34.6%	41.7%
<25 cm	68.7%	73.2%	74.9%	53.8%	60.1%	68.3%
<50 cm	90.1%	93.0%	92.2%	80.1%	87.6%	89.5%

## References

[1] Rishbeth H., Garriott O. (1969). Introduction to Ionospheric Physics.

[2] Böhm J., Salstein D., Alizadeh M.M., Wijaya D.D., Böhm J., Schuh H. (2013). Geodetic and Atmospheric Background. Atmopheric Effects in Space Geodesy.

[3] Klobuchar J.A. (1987). Ionospheric Time-Delay algorithm for Single-Frequency GPS Users. IEEE Trans. Aerosp. Electron. Syst..

[4] Van Dierendonck A.J., Russell S.S., Kopitzke E.R., Birnbaum M. (1978). The GPS Navigation Message. Navigation.

[5] Hochegger G., Nava B., Radicella S.M., Leitinger R. (2000). A family of ionospheric models for different uses. Phys. Chem. Earth Part C Sol. Terr. Planet. Sci..

[6] Radicella S.M., Leitinger R. (2001). The evolution of the DGR approach to model electron density profiles. Adv. Space Res..

[7] Di Giovanni G., Radicella S.M. (1990). An analytical model of the electron density profile in the ionosphere. Adv. Space Res..

[8] Nava B., Coïsson P., Radicella S.M. (2008). A new version of the NeQuick ionosphere electron density model. J. Atmos. Sol. Terr. Phys..

[9] Hoque M.M., Jakowski N., Osechas O., Berdermann J. Fast and improved ionospheric correction for Galileo mass market receivers. Proceedings of the 32nd International Technical Meeting of the Satellite Division of The Institute of Navigation (ION GNSS+ 2019).

[10] Johnston G., Riddell A., Hausler G., Teunissen P.J.G., Montenbruck O. (2017). The International GNSS Service. Springer Handbook of Global Navigation Satellite Systems.

[11] Jakowski N., Porsch F., Mayer G. (1994). Ionosphere-induced-ray-path bending effects in precise satellite positioning systems. Zeitschrift für Satellitengestützte Positionierung, Navigation und Kommunikation.

[12] Hoque M.M., Jakowski N. (2007). Higher order ionospheric effects in precise GNSS positioning. J. Geod..

[13] Aragon-Angel A., Hernandes-Pajares M., Defraigne P., Bergeot N., Prieto-Cerdeira R. Modelling and assessing ionospheric higher order terms for GNSS signals. Proceedings of the 28th International Technical Meeting of the ION Satellite Division, ION GNSS+ 2015.

[14] Rovira-Garcia A., Juan J.M., Sanz J. A Real-time World-wide Ionospheric Model for Single and Multi-frequency Precise Navigation. Proceedings of the 27th International Technical Meeting of the ION Satellite Division, ION GNSS+ 2014.

[15] Hernández-Pajares M., Roma-Dollase D., Garcia-Fernàndez M., Orus-Perez R., García-Rigo A. (2018). Precise ionospheric electron content monitoring from single-frequency GPS receivers. GPS Solut..

[16] Li Z., Wang N., Hernández-Pajares M., Yuan Y., Krankowski A., Liu A., Zha J., García-Rigo A., Roma-Dollase D., Yang H. (2020). IGS real-time service for global ionospheric total electron content modeling. J. Geod..

[17] Weber R., Boisits J., Joldzic N., Umnig E., Klug C., Thaler G., Karas R. (2016). Regiomontan-Regionale Ionosphärenmodellierung für Einfrequenz-Nutzer Anwendungen.

[18] Boisits J., Joldzic N., Weber R. (2016). Regional Ionospheric Modelling for Single-Frequency Users. Geophys. Res. Abstr..

[19] Böhm J., Böhm S., Boisits J., Girdiuk A., Gruber J., Hellerschied A., Krásná H., Landskron D., Madzak M., Mayer D. (2018). Vienna VLBI and Satellite Software (VieVS) for Geodesy and Astrometry. Publ. Astron. Soc. Pac..

[20] Zumberge J.F., Heflin M.B., Jefferson D.C., Watkins M.M., Webb F.H. (1997). Precise point positioning for the efficient and robust analysis of GPS data from large networks. J. Geophys. Res. Solid Earth.

[21] Lou Y., Zheng F., Gu S., Wang C., Guo H., Feng Y. (2016). Multi-GNSS precise point positioning with raw single-frequency and dual-frequency measurement models. GPS Solut..

[22] De Bakker P.F. (2016). On User Algorithms for GNSS Precise Point Positioning. Ph.D. Thesis.

[23] Shi C., Gu S., Lou Y., Ge M. (2012). An improved approach to model ionospheric delays for single-frequency Precise Point Positioning. Adv. Space Res..

[24] Ning Y., Han H., Zhang L. (2019). Single-frequency precise point positioning enhanced with multi-GNSS observations and global ionosphere maps. Meas. Sci. Technol..

[25] Aggrey J., Bisnath S. (2019). Improving GNSS PPP Convergence: The Case of Atmospheric-Constrained, Multi-GNSS PPP-AR. Sensors.

[26] Wang R., Gao J., Zheng N., Li Z., Yao Y., Zhao L., Wang Y. (2019). Research on Accelerating Single-Frequency Precise Point Positioning Convergence with Atmospheric Constraint. Appl. Sci..

[27] Zhou F., Dong D., Li W., Jiang X., Wickert J., Schuh H. (2018). GAMP: An open-source software of multi-GNSS precise point positioning using undifferenced and uncombined observations. GPS Solut..

[28] Cai C., Gong Y., Gao Y., Kuang C. (2017). An Approach to Speed up Single-Frequency PPP Convergence with Quad-Constellation GNSS and GIM. Sensors.

[29] Bassiri S., Hajj G. (1993). Higher-order ionospheric effects on the global positioning system observables and means of modeling them. Manuscripta Geod..

[30] Fritsche M., Dietrich R., Knöfel C., Rülke A., Vey S., Rothacher M., Steigenberger P. (2005). Impact of higher-order ionospheric terms on GPS estimates. Geophys. Res. Lett..

[31] Alizadeh M.M., Wijaya D.D., Hobiger T., Weber R., Schuh H., Böhm J., Schuh H. (2013). Ionospheric Effects on Microwave Signals. Atmopheric Effects in Space Geodesy.

[32] Hoque M.M., Jakowski N. (2012). A new global model for the ionospheric F2 peak height for radio wave propagation. Ann. Geophys..

[33] Schaer S. (1999). Mapping and predicting the Earth’s ionosphere using the Global Positioning System. Ph.D. Thesis.

[34] Schaer S., Gurtner W., Feltens J. IONEX: The IONosphere Map EXchange Format Version 1. Proceedings of the IGS AC Workshop.

[35] EPOSA—Echtzeit Positionierung Austria. http://www.eposa.at/.

[36] Banville S., Zhang W., Langley R.B. (2013). Monitoring the Ionosphere with Integer-Leveled GPS Measurements. GPS World.

[37] De Jonge P.J. (1998). A Processing Strategy for the Application of the GPS in Networks. Ph.D. Thesis.

[38] Teunissen P.J.G., Khodabandeh A. (2015). Review and principles of PPP-RTK methods. J. Geod..

[39] Kouba J. A Guide to Using INTERNATIONAL GNSS Service (IGS) Products. http://acc.igs.org/UsingIGSProductsVer21.pdf.

[40] Zhang H., Gao Z., Ge M., Niu X., Huang L., Tu R., Li X. (2013). On the Convergence of Ionospheric Constrained Precise Point Positioning (IC-PPP) Based on Undifferential Uncombined Raw GNSS Observations. Sensors.

[41] Odijk D., Zhang B., Khodabandeh A., Odolinski R., Teunissen P.J.G. (2016). On the estimability of parameters in undifferenced, uncombined GNSS network and PPP-RTK user models by means of S-system theory. J. Geod..

[42] Liu T., Wang J., Yu H., Cao X., Ge Y. (2018). A New Weighting Approach with Application to Ionospheric Delay Constraint for GPS/GALILEO Real-Time Precise Point Positioning. Appl. Sci..

[43] Dach R., Schaer S., Arnold D., Prange L., Sidorov D., Stebler P., Villiger A., Jäggi A. (2018). CODE Final Product Series for the IGS.

[44] Rebischung O., Schmid R. IGS14/igs14.atx: A new framework for the IGS products [poster]. Proceedings of the AGU Fall Meeting 2016.

[45] Landskron D., Böhm J. (2018). VMF3/GPT3: Refined Discrete and Empirical Troposphere Mapping Functions. J. Geod..

[46] Ihde J., Habrich H., Sacher M., Söhne W., Altamimi Z., Brockmann E., Bruyninx C., Caporali A., Dousa J., Fernandes R., Rizos C., Willis P. (2014). EUREF’s Contribution to National, European and Global Geodetic Infrastructures. Earth on the Edge: Science for a Sustainable Planet.

[47] Wu J.T., Wu S.C., Hajj G.A., Bertiger W., Lichten S. (1993). Effects of antenna orientation on GPS carrier phase. Manuscripta Geod..

[48] Petit G., Luzum B. (2010). IERS Conventions (2010). IERS Technical Note.

[49] Springer T., Hugentobler U. (2001). IGS ultra rapid products for (near-) real-time applications. Phys. Chem. Earth Part A Solid Earth Geod..

